# Emerging immunotherapy and tumor microenvironment for advanced sarcoma: a comprehensive review

**DOI:** 10.3389/fimmu.2025.1507870

**Published:** 2025-05-21

**Authors:** Hai Huang, Yiwei Fan, Shuling Zhang, Xueli Bai, Xiaonan Wang, Fengping Shan

**Affiliations:** ^1^ Department of Bone Oncology, The People’s Hospital of Liaoning Province, Shenyang, Liaoning, China; ^2^ Spine Surgery Unit, Shengjing Hospital of China Medical University, Shenyang, Liaoning, China; ^3^ Department of Oncology, Shengjing Hospital of China Medical University, Shenyang, Liaoning, China; ^4^ Department of Gynecology, The Fourth Affiliated Hospital of China Medical University, Shenyang, Liaoning, China; ^5^ Department of Immunology, College of Basic Medical Science, China Medical University, Shenyang, Liaoning, China

**Keywords:** sarcoma, immunotherapy, adoptive T cell therapy, vaccine, immune checkpoint inhibitors

## Abstract

Sarcomas are heterogeneous mesenchymal malignancies classified as soft-tissue sarcomas (STS) and bone sarcomas. Advanced cases respond poorly to standard therapies, highlighting the need for novel strategies. Immunotherapies, including PD-1/PD-L1 inhibitors, adoptive cellular therapies, vaccines, and oncolytic viruses, have shown promise in specific sarcoma subtypes. This review explores these approaches, emphasizing the prognostic significance of immune cells within the tumor microenvironment (TME), such as tumor-associated macrophages (TAMs) and tumor-infiltrating lymphocytes (TILs), and their correlation with clinical outcomes. We also discuss challenges in immunotherapy efficacy, the importance of biomarker-driven personalized therapies, and the potential of a combination regimen with chemotherapy, radiation, and cytokine agents. Overall, this review highlights the evolving role of immunotherapy in advanced sarcomas, the critical influence of the TME, and the need to optimize synergistic treatment approaches to enhance patient outcomes.

## Introduction

Sarcomas are heterogeneous malignancies arising from mesenchymal precursors encompassing bone, cartilage, fat, and muscle. Although rare in adults, comprising about 1% of global cancer diagnoses, sarcomas account for nearly 15% of pediatric malignancies (1). The World Health Organization recognizes over 70 subtypes, typically classified into two primary categories: soft-tissue sarcoma (STS) and bone sarcoma, each with unique biology and clinical behaviors (2, 3). For localized disease, the current standard treatment is surgical resection often combined with radiotherapy. Nevertheless, approximately half of patients with high-grade tumors later develop metastasis, yielding a median overall survival (OS) of 14–19 months (4). For unresectable or advanced cohorts, standard first-line systemic therapy is doxorubicin alone or in combination with the alkylating drug ifosfamide (5). Second-line settings, such as novel chemotherapeutic agents trabectedin (6), eribulin (7), and tyrosine kinase inhibitors (TKIs) like pazopanib (8) are useful therapy options for specific sarcoma subtypes. Notably, their significance for enhancing OS remains uncertain. These therapies only yield limited durable response, with objective response rates (ORR) of 10-20% and median progression-free survival (PFS) of 4 months, highlighting the crucial need for more effective treatment options.

## Specific challenges in sarcoma treatment

Immunotherapies, especially immune checkpoint inhibitors (ICIs), have achieved considerable benefit in certain sarcoma subtypes. Multiple trials are investigating ICI combinations with other therapies. However, specific challenges remain due to sarcoma heterogeneity, limited targetable antigens, and a lack of subtype-specific trials. ICIs generally demonstrate lower efficacy in sarcomas than in other solid tumors, with basket trials often grouping diverse subtypes, which complicates the identification of effective treatments for rare forms (9). Additionally, immunosuppressive tumor microenvironments (TME), low tumor mutational burdens (TMB), and weak immunogenicity of tumor-associated antigens (TAAs) hinder treatment success. Despite these limitations, combining ICI with other medications has shown promising synergistic advantages. Emerging approaches, such as adoptive cell transfer and oncolytic viruses (OVs), offer new opportunities to address these challenges and may enhance therapeutic outcomes for sarcoma patients.

**Figure 1:**
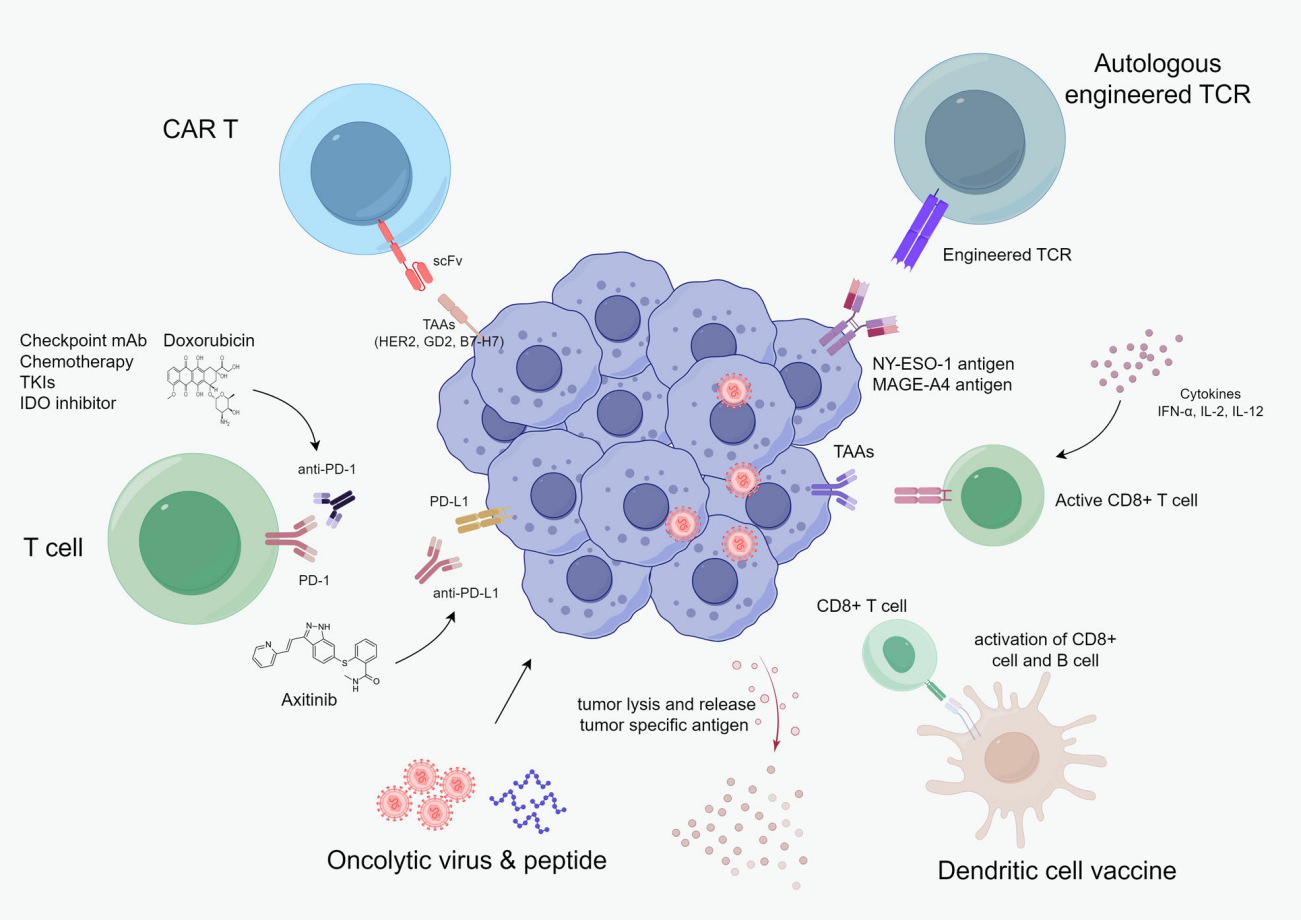
Current cancer immunotherapy landscape in advanced sarcoma, including checkpoint monoclonal antibodies, chemotherapy, TKI, adoptive cellular therapy, oncolytic virus & peptide, vaccine, and cytokine-based therapy.

This article reviews the current evidence supporting the utility of immunotherapy in advanced sarcoma, as well as the existing immunotherapy strategies (**Figure 1**), including anti-PD-1/PD-L1 therapy, adoptive T-cell therapy (ACT), vaccines, oncolytic virus, and cytokine-based immunotherapy.

## Immunological characteristic in different sarcoma subtype

Sarcomas are generally considered “immunological quiet”, characterized by a low TMB, immunosuppressive TME, reduced T-cell infiltration, and increased HIF-1α, macrophages, and neutrophils. However, certain subtypes like alveolar soft-part sarcoma (ASPS), synovial sarcoma (SS), and undifferentiated pleomorphic sarcoma (UPS) display an immunologically “hot” phenotype: higher TMB, elevated PD-L1 expression, and presence of tertiary lymphoid structures (TLS), correlating with improved responses to checkpoint inhibitors (10).

The sarcoma TME comprises immune cells, stromal cells (including cancer-associated fibroblast, CAF), and endothelial cells. These components interact with tumor cells, influencing progression, immunotherapy response, and clinical outcomes. The specific immune contexture in sarcoma is often marked by predominate tumor-associated macrophages (TAM), dysfunctional tumor-infiltrating lymphocytes (TIL) with reduced CD8+ T and NK cell activity, increased regulatory T cells (Tregs), limited B cells, and impaired dendritic cell (DC) function.

### TAMs

TAMs are abundant myeloid cells in TME that contribute to immune suppression, angiogenesis, and metastasis. They present antigens via surface MHC molecules and secret immunomodulatory cytokines, fostering a pro-tumoral milieu and enhancing vascular remodeling (11). In sarcomas, TAM densities often exceed those of TILs (12–14). These macrophages predominantly display an M2-type (immunosuppressive), marked by high expression of SIRPα, CD47, CD68, CD163, and CSF1R (14, 15–17), which collectively promote phagocytosis resistance and immunosuppression.

Clinically, high TAM infiltration predicts poorer outcomes in both soft−tissue and bone sarcomas (14, 18–20). In a cohort of 188 STS patients, high TAM levels were independently associated with an increased risk of local recurrence (20). In 75 sarcoma specimens, greater numbers of CD163+/CD204+ macrophages at tumor margins correlated with reduced disease−free survival (18). Similarly, undifferentiated leiomyosarcomas with dense CD163^+^/CD68^+^ infiltration exhibited worse overall survival (19). In chondrosarcoma, CD68+ CD163+ TAMs are the main immune population (21), and a high CD68+/CD8+ ratio independently forecasts metastatic presentation and poor prognosis.

### TILs

STS generally have fewer TILs and exhibit lower CD4+/CD8+ ratios compared to other immunoreactive cancer (22). High-grade sarcoma, such as leiomyosarcoma, exhibits higher infiltration of CD3+, CD8+, and FOXP3+ T-cells (13), while infiltrating CD20+ B-cells, though rarely detected in STS, are correlated to improved outcomes (23).

### CD8+ T cells

CD8+ T cells are critical for antitumor immunity but become dysfunctional in sarcoma through upregulation of inhibitory receptors. In the primary UPS cohort, approximately one-third of cases exhibit high CD3+/CD8+ densities associated with favorable survival outcomes (24). In SS, greater CD8+ or FOXP3+ infiltration corresponds to improved OS (25). However, in angiosarcoma, neither PD-1/PD-L1 expression nor CD8+ levels predict outcomes, underscoring the subtype-specific complexity (26). Although gastrointestinal stromal tumor (GIST), myxofibrosarcoma, and pleomorphic sarcoma all feature high CD8+ densities, but lack of co−stimulatory ligands in GIST limits cytotoxic efficacy (27).

Correlative biomarker studies highlight that combined immune parameters best predict clinical benefit. In the SARC028 trial, pre−treatment densities of activated CD8+ T cells and PD-L1+ TAMs correlated with pembrolizumab response (28). Co-presence of CD8+ TILs and neoantigens further improved survival compared to either factor alone (29). Likewise, trials of interleukin-2 pathway agonists plus nivolumab demonstrated that CD8+ infiltration together with PD-1 expression correlated with increased ORR (23, 30).

### Tregs

Tregs facilitate tumor immune evasion by secreting IL-10 and TGF-β, expressing CTLA-4 and PD-1, and thereby inhibiting effector T cell responses. Treg infiltration varies across sarcoma subtypes, with GIST showing the highest density of FOXP3+ density (36%) among sarcomas (12). The prognostic significance of Tregs in STS is undefined due to the lack of sample number and heterogeneity. In one cohort of 163 STS samples, 11.7% were PD-L1 positive and 25.2% showed high FOXP3+ infiltration, both metrics independently predicted poor prognosis in multivariate analysis (31). Another study linked high Treg levels with increased local recurrence risk, regardless of surgical margin status (32).

## Biomarkers of immunotherapy response

### TMB and dMMR

TMB correlates with ICI efficacy, as higher TMB generates more neoantigen, enhancing immune recognition (33). The FDA defines TMB-high as ≥ 10 mutations per megabase (Mb), which predicts a stronger response to PD-1 blockade (34). However, most STS subtypes exhibit median TMB below three mutations per Mb, and only ~2% qualify as TMB-high, making it an impractical biomarker in STS (35, 36). Analysis of 1,407 sarcomas in the GENIE database confirmed low TMB across sarcoma categories, with TMB-high in 3.8% of STS and 0.6% of bone sarcomas (37). Specific histologies (e.g., angiosarcoma) exhibit higher TMB, with 63.4% of aggressive cases meeting the high threshold (38, 39). Conversely, translocation-driven subtypes (e.g., SS) typically display low TMB, though rare high-TMB cases (~6.3%) have been reported (40).

A phase II trial (NCT02834013) of ipilimumab and nivolumab in angiosarcoma reported a 25% ORR, rising to 60% in cutaneous scalp and face tumors (41). Among seven TMB-evaluated patients, only the single TMB-high case responded. Similarly, PD-1 blockade benefits patients with cutaneous head and neck angiosarcomas, regardless of TMB elsewhere, suggesting anatomical context may outweigh mutational burden (42).

MMR deficiency (dMMR) machinery is associated with high mutational load and predicts PD-1 blockade efficacy (39). However, dMMR remains exceptionally rare (~1%) in STS, further constraining its role as a predictive biomarker.

### PD-L1 expression

Unlike many carcinomas, PD-L1 levels in sarcomas are highly variable and only modestly predictive of ICI response. Immunohistochemistry studies report PD−L1 positivity in 12-23% of STS cases, depending on the subtype and antibody used (12, 43). In SARC028, a higher density of PD-L1+TAMs and infiltrating CD8+ T cell correlated with ICB response; nonetheless, only 2 of 40 PD-L1+ sarcomas responded (28). PD-L1 expression often increases as the tumor progresses and generally portends a worse prognosis. One small SS series found lower PD-L1 in recurrent versus diagnostic specimens (44). Radiotherapy can increase PD-L1 expression when given preoperatively (45). Overall, dynamic and context−dependent PD-L1 regulation in the sarcoma microenvironment limits its reliability as a stand−alone biomarker.

### B cell and TLS

B cell subtypes in the TME play dual roles: antigen-presenting B cells within TLS activate CD4+ and CD8+ T cells to mount antitumor responses (46–48), whereas regulatory B cells secrete immunosuppressive cytokines (49, 50). High-immune-infiltrate STS samples are enriched for B-cell-rich TLS, which strongly predicts better response rates and survival in pembrolizumab-treated cohorts. TLS are organized immune cell clusters that resemble secondary lymphoid organs. They play a critical role in generating delayed immune response by recruiting TILs. The spatial structure of TLS, including germinal centers, cellular composition, and tumor location affects patient prognosis (51).

Transcriptomic profiling of STS has defined five immune phenotypes, including immune-low, immune-high, and highly vascularized groups (23). The immune-high/TLS-rich class exhibited superior survival and a 30% ORR in Phase II pembrolizumab trials, compared to 2.4% overall (52). Similar TLS−related benefits have been observed in melanoma, RCC, and other ICI-treated malignancies, highlighting their potential as pan-cancer predictors of ICB response (53–55).

### IDO

Indoleamine 2,3-dioxygenase (IDO) catalyzes tryptophan to kynurenine, creating an immunosuppressive niche that impairs effector T cells and promotes Tregs activation (56, 57). In a study of 371 primary STS patients, IDO was detected on endothelial cells in 23% and on tumor cells in 41%; 56% of samples exhibited elevated kynurenine (58). Higher kynurenine levels, but not PD-L1 expression, are associated with worse OS. A phase II trial of pembrolizumab in selected STS subtypes achieved only a 6% partial response. The reason was likely due to high baseline infiltration of IDO1+ macrophages (59). The treatment further increased the plasma kynurenine/tryptophan ratio, suggesting the IDO1-mediated immunosuppression as a resistance mechanism.

Although combining the IDO1 inhibitor epacadostat with pembrolizumab showed early promise in melanoma, the subsequent phase III KEYNOTE-252 trial failed (60), possibly due to compensatory upregulation of TDO or IDO2. A small phase II study using the same combination in sarcoma also yielded limited benefit (61). These results emphasized the need to elucidate the IDO/kynurenine signal in the sarcoma TME and to refine dosing and combinatorial strategies before deploying IDO pathway inhibitors as clinical biomarkers or therapeutic partners.

## Immune checkpoint blockage for advanced sarcomas

### ICI monotherapy or combined with other ICIs

Early trials using ICI monotherapy demonstrate unsatisfactory activity (62, 63). In the pioneering phase II SARC028 trial, 84 STS patients treated with pembrolizumab had an ORR of 18%. The best activity was observed in UPS of 40% and dedifferentiated liposarcoma cohorts of 20%, while osteosarcoma showed only a 5% response (64). A subsequent basket trial for rare sarcomas reported an ORR of 6.2% in the pembrolizumab monotherapy group, with no complete responses (65). Pooled analyses of anti-PD1/PD-L1 therapy in advanced STS showed an ORR of 15.1% and a non-progression rate (NPR) of 58.5% (66). The UPS and ASPS displayed the best response, while leiomyosarcoma and osteosarcoma had the lowest response rates. Nivolumab alone showed similarly modest activity, with a 12% response rate in certain sarcoma subtypes. A combination of nivolumab and CTLA-4 inhibitor ipilimumab demonstrated an improved response. In the Alliance A091401 trial for metastatic sarcoma, the combination therapy had a 16% ORR compared to 5% for nivolumab alone (67, 68) (**Table 1**).

**Table 1:** Key clinical trials of ICI monotherapy or combination in sarcomas.

NCT number	Clinical Trial	Phase	Study agent/combination	Sarcoma subtype/ evaluable patients	ORR (%)	Outcomes/details
ICI monotherapy or combination with other ICI						
NCT02301039	Tawbi et al. SARC028, 2017 (64)	Phase II	pembrolizumab	40 STS cohort; 40 BS cohort	18% in STS 5% in BS	STS patients, PFS: 18 weeks; OS: 49weeks. BS patients, PFS: 8 weeks; OS: 52weeks.
NCT03012620	Blay et al. AcSé Pembrolizumab, 2023 (65)	Phase II (basket trial)	pembrolizumab	98 rare STS (34 chordoma, 14 ASPS, 12 SMARCA4-deficient, 8 DSCRT, 31 others)	6.2% at week 12	PFS 2.75 ms; OS 19.7 ms
NCT02500797	D’Angelo et al. Alliance A091401, 2018 (68)	Phase II	nivolumab plus ipilimumab vs nivolumab	42 sarcomas (3 AS, 4 BS, 14 LMS, 2 LPS, 6 SCS, 2 SS, 6 UPS/MFH, 1 unspecified, 4 others); 43 sarcomas (5 BS, 15 LMS, 3 LPS, 2 unspecified, 5 SCS, 2 SS, 5 UPS, 6 others)	16% vs 5%	PFS: 4.1 ms vs 1.7 ms, OS: 14.3 ms vs 10.7 ms
NCT03307616	Roland et al., 2024 (69)	Phase II	neoadjuvant nivolumab or nivolumab/ipilimumab	17 DDLPS and 10 UPS	pathologic response was 8.8% in DDLPS and 89% in UPS	24-month relapse-free survival was 38% in DDLPS and 78% in UPS
NCT03141684	Chen et al., 2023 (70)	Phase II	atezolizumab	52 ASPS	37%	PFS: 20.8 ms
ICIs combination with chemotherapy or TKI						
NCT02888665	Pollack et al., 2020 (71)	Phase I/II	pembrolizumab plus doxorubicin	37 anthracycline-naive sarcoma (11 LMS and others)	13% for phase II patients and 19% overall	PFS: 8.1ms, OS: 27.6 ms
N/A	Livingston et al., 2021 (72)	Phase II	pembrolizumab plus doxorubicin	30 STS	36.7%	PFS: 5.7 ms; OS: 17 ms
N/A	Reichard et al. NITRA-SARC, 2023 (73)	Phase II	nivolumab plus trabectedin	Group A-lipo- or leiomyosarcomas: 43 STS (28 LMS and 15 LPS); Group B-non-L-sarcomas: 49 STS (12 UPS, 11 SCS, 6 FMS, 5 SS, 4 EpS)	overall PFS rate 6-months: 47.6% vs 14.6%	PFS: 5.5 ms vs 2.3 ms; OS: 18.7 ms vs 5.6 ms
NCT03138161	Gordon et al. SAINT, 2023 (74)	Phase I/II	nivolumab/ipilimumab plus trabectedin	26 LMS, 14 LPS, 9 UPS, 7 RMS, 5 SS, 24 others	6 CR, 14 PR, 49 SD, 25.3% best response rate	PFS: 6.7ms, OS: 24.6 ms
NCT03899805	Haddox et al., 2024 (75)	Phase II	pembrolizumab plus eribulin	57 STS (19 LMS, 20 LPS, 18 UPS/other)	2 PR in LMS cohort; 3 PR in LPS; 1 CR and 5 PR in other cohort	12 week PFS rate was 36.8% for LMS, 69.6% for LPS, and 52.6% for UPS/other
NCT02636725	Wilky et al., 2019 (76)	Phase II	Pembrolizumab plus axitinib	33 STS (12 ASPS, 6 LMS, 5 High-grade PS, 2 DDLPS, 8 other histotypes)	The overall 3-month PFS rate: 65·6%	PFS: 4.7 ms; OS: 18.7 ms
NCT03277924	Martin-Broto et al. IMMUNOSARC 2020 (77)	Phase I/II	nivolumab + sunitinib	52 STS (9 SS, 8 UPS, 7 clear cell sarcoma, 7 SFT, 7 EpS, 5 AS, 4 ESMCS, 4 ASPS, 1 EHET)	the 6-month PFS rate: 48%	PFS: 5.6 ms; mOS: 24 ms
N/A	Liu et al., 2022 (78)	Phase II	benmelstobart (anti-PD-L1) plus anlotinib	30 STS (12 ASPS, 7 SS, 5 UPS, 4 LMS, 2 others)	36.6%	PFS: 7.8 ms; OS: not reached
NCT03798106	Cho et al., 2024 (55)	Phase II	durvalumab plus pazopanib	47 STS (12 LMS, 5 MPNST, 4 SS, 4 MFS, 4 UPS, 4 DSRCT, 14 others)	30.4%	PFS 7.7 ms, 1-year OS of 71.7%

OS, Overall survival; PFS, Progression-free survival; STS, soft tissue sarcoma; BS, bone sarcoma; NPR, non-progression rate; N/A, unmentioned.

Neoadjuvant nivolumab or ipilimumab also showed significant efficacy in resectable high−grade sarcomas. In retroperitoneal dedifferentiated liposarcoma (DDLPS) and UPS, 89% of resected specimens showed pathologic response (69). The two-year OS rate exceeded 80% in both cohort and heightened intratumoral B cell infiltration correlated with superior survival. Similarly, a phase II study in classical Kaposi sarcoma (cKS) achieved an 87% response rate (6/13 evaluable patients) with combined nivolumab and low-dose ipilimumab (79).

Emerging novel PD−1/PD−L1 inhibitors have shown promising efficacy in ASPS. Toripalimab, a high-affinity anti-PD-1 antibody, demonstrated a 25.0% ORR in a phase I trial of advanced ASPS, with a median PFS of 11.1 months and OS of 34.7 months (80). Atezolizumab, an anti-PD-L1 agent, achieved a 37% ORR and 20.8 months median PFS in a phase II ASPS cohort (70). Though combination strategies with CTLA−4 blockade increased toxicity, pharmacodynamic analyses indicated that even tumors lacking baseline PD-1/PD-L1 expression may convert to an ICI-responsive phenotype during treatment. These data affirm that dual−checkpoint approaches and novel agents can overcome inherent sarcoma resistance, but also underscore the need for refined biomarker−driven patient selection.

### Combination of ICIs plus chemotherapy

Combination regimens of ICIs and chemotherapy have demonstrated promising results in advanced sarcoma, particularly in anthracycline−naive and high−grade subtypes. In the first phase 1/2 trial combining pembrolizumab with doxorubicin in anthracycline-naive sarcoma, the study did not meet its primary endpoint for ORR (19% overall), but achieved a PFS of 8.1 months and OS of 27.6 months, both favorably compared with prior studies (71). The following phase II trial in unresectable STS confirmed the combination’s manageable safety profile, reporting a 36.7% ORR and an 80.0% disease control rate (DCR) (72). PD-L1 expression was linked to improved ORR, but not to PFS or OS.

Alkylating agent metronomic cyclophosphamide has also been combined with PD-1 inhibitors. Despite strong preclinical synergy, a phase II trial showed limited activity in STS, possibly owing to IDO1-expressing TAMs (59). Combining IDO inhibition with pembrolizumab yielded only a 3.3% ORR at 24 weeks (61).

Trabectedin is a marine-derived alkylating agent approved for anthracycline-resistant liposarcoma or leiomyosarcoma. It can destroy cancer cells and expose tumor neoantigens to immune recognition. Trabectedin combined with low-dose cyclophosphamide modulates macrophage polarization in the sarcoma microenvironment, reducing M2 macrophages and increasing CD8+ T cells that correlated with improved prognosis (81, 82). In a cohort of 92 patients, trabectedin plus nivolumab extended median PFS to 9.8 months versus 4.4 months, and OS to 24.6 months versus 13.9 months compared to earlier data (73). First−line regimens combining trabectedin with ipilimumab/nivolumab achieved a best response of 25.3% and an 87.3% DCR (74). A seven−year follow−up confirmed durable safety and efficacy, with 25% of participants alive at the study cutoff (83).

Finally, eribulin, a microtubule-binding agent, activates the cGAS-STING signal and remodels immune infiltration. In combination with pembrolizumab, eribulin produced a 12-month PFS rate of 36.8% in leiomyosarcoma, 69.6% in liposarcoma, and 52.6% in UPS (75). High serum levels of IL-4 and IFN-α were linked to therapeutic benefits. Collectively, these studies underscore the potential of chemo−immunotherapy combinations to convert immunologically “cold” sarcomas into more responsive tumors, warranting further biomarker−driven optimization.

### Combination of ICI and TKI target therapy

Combining ICIs with anti-angiogenic TKIs has shown synergistic effects in treating advanced sarcoma. This synergy is partly attributed to the normalization of tumor vasculature, which enhances the effector cell infiltration, and converts the suppressive TME into an active state (84).

ASPS, a rare subtype resistant to cytotoxic therapy, often harbors the *ASPSCR1-TFE3* fusion, leading to upregulation of HIF-1α and VEGF. TKI has been the most active option for ASPS, whereas the majority could develop resistance. A phase II trial combining pembrolizumab with the VEGF inhibitor axitinib in advanced sarcoma reported an ORR of 25% and a median PFS of 4.7 months (76). Notably, ASPS patients achieved an ORR of 54.5% and a median PFS of 12.4 months. The observed outcomes in ASPS likely reflect the contribution of PD-1 blockade, as axitinib monotherapy yielded no responses in four ASPS.

Sunitinib can activate immune cell subsets, inducing IFN-γ-producing effector T cells via DCs, and synergize with PD-1 blockade (85). Sunitinib plus nivolumab (ImmunoSarc-I trail) demonstrated an ORR of 21% and an 18-month OS rate of 100% (77). Due to high toxicity, this regimen used a lower dose of sunitinib. A subsequent phase II trial (NCT03277924) showed potential activity in several other subtypes (86, 87). Angiosarcoma patients exhibited a higher efficacy, with a PR rate of 33%, and a median PFS of 3.93 months.

Anlotinib is a multi-kinase angiogenetic inhibitor that is recommended as a first-line treatment for metastatic ASPS (88). A combination of anlotinib and novel PD-L1 antibody TQB2450 (Benmelstobart) exhibited a favorable efficacy in metastatic STS patients unresponsive to chemotherapy (78). Among the 30 enrolled patients, the ORR was 36.7%, and median PFS was 7.8 months. In an expanded ASPS cohort, this combination showed an ORR of 79.3%, including 3 CR and 20 PR (89). TLS emerged as a potential predictive marker for immunotherapy efficacy in ASPS.

A phase II trial (NCT03798106) combining durvalumab with pazopanib in metastatic STS met its pre-specified endpoint (55). The ORR was 30.4% and mPFS was 7.7 months. High CD20+ B cell infiltration and vessel density led to a longer PFS and improved response. Infiltrated CD20+ B cell was identified as an independent predictor of PFS.

To summarize, these combined regimens of anti-angiogenic inhibitors and ICIs demonstrate synergistic anti-tumor effects and promising activity in patients with ASPS and some vascular subtypes. However, all these researches are limited by inadequate sample sizes and the absence of controlled arms, which restrict the ability to identify molecular markers of response. Further investigations with larger, well-designed trials are required to validate these findings and explore predictive biomarkers.

### Real-world efficacy of ICI in advanced sarcoma

Several retrospective studies have reported the real-world efficacy of immunotherapy, in advanced STS, either alone or combined with anti-angiogenic therapy (90–93). These studies reported variable outcomes, influenced by patient characteristics and sarcoma subtypes. Liu et al. (90) reported a 19.4% ORR with pembrolizumab monotherapy in advanced STS, alongside a median PFS of 2.9 months and OS of 12.0 months. Although certain subtypes like ASPS and UPS are considered more responsive to ICIs, real-world data remain limited due to patients’ poorer health status and extensive prior treatment. Notably, treatment duration and ECOG performance status were independent predictors of PFS and OS. In another study, Nasr et al. assessed the ICI efficacy in metastatic UPS and other high-grade pleomorphic sarcomas (91). The median PFS was 2.9 months, with a 6-month PFS of 32%, and a median OS of 12.9 months. Prior radiotherapy and ICI type was independently associated with PFS. These findings align with the broader literature, suggesting the ICI effectiveness in advanced STS.

Combining ICIs with anti-angiogenic therapy has shown enhanced efficacy. One real-world study (93) reported an ORR of 48.1% and a median PFS of 8.9 months with such a combination. ASPS cohorts exhibited a higher PR rate (71.4%), indicating significant benefit. Another analysis (92) confirmed the potential of this combination, reporting an ORR of 17.6% and a median PFS of 5.8 months. Patients with ASPS or clear cell sarcoma (CCS) had significantly longer median PFS (16.2 months) compared to other subtypes (4.4 months). Multivariate analyses identified ECOG status and treatment line as key predictors of both PFS and OS.

Collectively, combined ICIs with anti-angiogenic therapy offer promising clinical benefits for STS, particularly in subtypes like ASPS and CCS. However, response rates vary due to patient characteristics and treatment history. Factors like ECOG status and treatment timing significantly influence outcomes. Challenges remain in optimizing therapy sequencing, understanding synergetic mechanisms, and tailoring strategies to specific patient subsets. Current decisions often rely on clinical experience, emphasizing the need for larger prospective studies and biomarker identification to address STS heterogeneity and improve treatment outcomes.

## Adoptive T-cell therapy

ACT represents a promising immunotherapeutic strategy that harnesses engineered T cells to target TAAs expressed by cancer cells. Three classical ACTs methods are clinically developed, TCR-T, CAR-T, and TILs.

### TCR-T

Letetresgene autoleucel (lete-cel) is an autologous engineered TCR therapy targeting NY-ESO-1 antigen and specific HLA-A*02 alleles. In a phase II trial (NCT03967223) (94), 87 patients with metastatic SS or myxoid round cell liposarcoma (MRCLS) expressing NY-ESO-1 were treated with lete-cel at doses of 1-15×10^9^ transduced cells. The ORR was 39% for SS and 41% for MRCLS, with a median response duration of 10.6 months. Another phase I trial (NCT04318964) reported a novel TCR-T therapy targeting NY-ESO-1 in sarcoma (95). Twelve patients received cell infusions and low doses of IL-2 injection post-adoptive transfer. The ORR was 41.7%, with a median PFS of 7.2 months and a median duration of 13.1 months. The regimen exhibited favorable efficacy and safety profiles.

Afamitresgene autoleucel (afami-cel), an autologous TCR-T product targeting MAGE-A4 in HLA-A*02-positive patients. In the phase II SPEARHEAD-1 trial, afami-cel achieved an ORR of 43.2% with a median response duration of six months in patients with unresectable or metastatic SS who had received prior chemotherapy (96). Common adverse events included cytokine release syndrome, nausea, and fatigue. Afami-cel received accelerated approval from the U.S. FDA, making it the first TCR-based cell therapy for rare sarcoma subtypes (97).

Although TCR-based therapies targeting cancer-testis antigens (CTAs) show initial responses, many patients eventually experience disease progression. Future research should focus on understanding resistance mechanisms, overcoming HLA restrictions, and expanding the repertoire of targetable TAA.

### CAR-T

CAR-T cell therapy has shown remarkable success in hematological malignancy, but faces challenges in solid tumors, including sarcomas, due to issues with T-cell trafficking, tumor heterogeneity, and the immunosuppressive TME. Current strategies aim to improve long-term efficacy by targeting conserved antigens that minimize toxicity to healthy tissues and enhancing CAR-T cell homing and persistence.

### B7-H3

B7-H3 (CD276) is overexpressed in many pediatric solid tumors including pediatric sarcoma and neuroblastoma, with limited expression in normal tissue. In an analysis of 156 sarcoma specimens, 91% exhibited B7-H3 expression, with 61% showing high levels (98).

Clinical trials of B7-H3 targeted therapies, such as MGA271 (Fc-optimized humanized anti-B7H3 mAb) and MGC018 (B7-H3 ADC), have demonstrated antitumor activity but also raised concerns about toxicity (99).

B7-H3 CAR-T showed safety and good tolerability in early-phase trials for relapsed pediatric cancers, but limited efficacy (100, 101). The STRIvE-02 trial (NCT04483778) (102) reported no objective responses (n=9) after initial infusions. However, a single patient achieved a response following a second infusion, possibly due to prior radiation therapy enhancing CAR-T cell expansion. Combining radiation with CAR-T may modulate the TME to improve outcomes. Ongoing studies are exploring bispecific B7-H3xCD19 CAR-T cells and combinations with PD-1 inhibitors to enhance efficacy (103, 104).

To improve CAR-T cell homing, strategies involve engineering cells to express chemokine receptors like CXCR2 and CXCR6n. Preclinical models have shown that CXCR2-modified B7-H3 CAR-T cells exhibit enhanced trafficking to osteosarcoma sites and improved cytolytic activity, leading to prolonged survival (105).

### HER2

HER2 CAR-T cell therapy has demonstrated safety in advanced sarcomas, but limited efficacy due to poor CAR-T cell expansion and persistence. The HEROS 2.0 phase I trial allowed multiple HER2 CAR-T infusions, resulting in a 50% (7/14) clinical benefit (106). Three patients (21%) achieved complete remission, including one with long-term remission. Current studies are exploring combinations of HER2 CAR-T with ICIs like pembrolizumab or nivolumab (NCT04995003) to enhance CAR-T expansion and efficacy.

### GD2

GD2 is highly expressed in neuroblastoma and pediatric tumors like Ewing sarcoma and osteosarcoma but minimally in normal tissues. GD2-targeting antibodies and GD2-targeted CAR-T cells have shown promising activity in relapsed neuroblastoma (107, 108). A phase I trial (NCT02107963) of third-generation GD2 CAR-T cells demonstrated feasibility and safety in osteosarcoma and neuroblastoma patients, but limited efficacy (109). Multi-omic analyses indicated that baseline CXCR3+ monocytes correlated with improved CAR T cell expansion, suggesting the peripheral immune environment influences therapy efficacy. Further research is needed to clarify myeloid-driven resistance mechanisms and enhance GD2 CAR-T cell efficacy in pediatric sarcoma.

Collectively, CAR-T therapy holds promise for treating solid tumors like sarcomas, despite the challenges exist. Strategies incorporating chemokine receptor modification, targeting tumor stroma, combination therapies, cytokine support, and metabolic reprogramming are being explored to enhance CAR-T cell persistence and antitumor activity (110). Continued research and clinical trials are essential to optimize these approaches and improve outcomes for sarcoma patients.

## TILs therapy in sarcoma

TILs are immune cells within tumors capable of recognizing various cancer-associated antigens. In metastatic melanoma, TIL therapy has shown an ORR of 30-60% (111–113). For advanced STS patients, several ongoing trials are exploring TIL monotherapy or combination strategies, but fewer efficacy were reported (114, 115).

## Oncolytic virus therapy

OVs therapy represents a novel immunotherapeutic strategy that utilizes natural or genetically engineered viruses to selectively infect and lyse cancer cell. This approach remodels the TME, enhances tumor immunogenicity, and can sensitize tumors to other immunotherapies. Talimogene laherparepvec (T-VEC), an oncolytic HSV-1 virus, is currently approved for treating recurrent, unresectable melanoma. In STS, several OVs have demonstrated efficacy in preclinical models, but few have advanced to clinical trials. A phase II trial combining the oncolytic vaccinia virus JX-594 with low-dose cyclophosphamide in advanced STS showed no clinical benefit, as all patients experienced disease progressing within six months (116). Adding the PD-L1 inhibitor avelumab provided limited additional benefit (117), with only one angiosarcoma patient achieving a PR.

The success of T-VEC in melanoma has prompted investigations into its potential in sarcomas. A Phase Ib trial combining T-VEC with preoperative radiation in locally advanced STS demonstrated tolerability but limited efficacy, with only 5 of 23 patients achieving pathological CR (118). In a Phase II trial, T-VEC combined with pembrolizumab showed strong antitumor activity across sarcoma subtypes, achieving a 30% ORR at 24 weeks, with notable responses in angiosarcoma, where 71% of patients achieved PR (119, 120). OH2 is an oncolytic HSV-2 expressing GM-CSF. A combination of OH2 and anti-PD-1 therapy achieved a 16.7% ORR, with one CR and durable disease control in angiosarcoma (121).

Oncolytic peptides, such as LTX-315, offer a non-viral approach to oncolysis by triggering anticancer immunity through remodeling the TME (122). A pilot trial evaluated LTX-315 in six heavily pretreated sarcoma patients, with four proceeding to adoptive cellular therapy (114). The treatment exhibited manageable toxicity and induced systemic immune responses, leading to disease stabilization in some patients. The best clinical response was a long-lasting stable disease in one patient, with tumor-reactive T cells detected in the blood. Further optimization is needed to improve clinical benefit. Subsequent studies found that LTX-315 triggers anticancer immunity by promoting MyD88-dependent DC maturation (115).

## Vaccine therapy

Vaccine-based immunotherapy strategies are gaining traction in sarcoma, particularly those targeting cancer-testis antigens like NY-ESO-1. Personalized peptide vaccines have shown potential. A phase II trial reported a median OS of 9.6 months in refractory sarcoma patients, slightly exceeding the 8-month OS for second-line palliative chemotherapy. Some cases experienced lung metastasis reduction and prolonged stable disease (123). Combining long peptide antigen (LPA) vaccines with TCR-T has shown efficacy in - preclinical models resistant to checkpoint inhibitors (124). A phase I trial combining NY-ESO-1-specific TCR-T cells with a lymph node-targeted LPA vaccine in refractory SS showed durable tumor shrinkage lasting over two years and sustained TCR-T cell persistence in one patient (125).

DC vaccination could enhance antitumor immunity by inducing CD8+ T-cell responses and has shown promise (126). In a phase I/II study of 35 advanced sarcoma patients, only one exhibited a PR, six had stable disease, and increased IFN-γ and IL-12 levels were observed post-treatment (127). Case studies also highlight durable responses, such as a pediatric Ewing’s sarcoma patient surviving beyond two years post-DC vaccination, and a refractory SS patient showing over 2.5 years of disease control with NY-ESO-1-targeting lentiviral DC therapy (128, 129). DC vaccines can also enhance CAR-T cell therapy by improving persistence, tumor infiltration, and adaptive immune activation, and thereby leading to increased tumor cytolysis (128, 130). Combining DC vaccines with adoptive cellular therapy or ICIs offers potential strategies to amplify antitumor efficacy, warranting further exploration in sarcoma treatment.

CMB305 is a lentiviral-based prime-boost vaccine targeting NY-ESO-1. In a phase Ib study of 79 sarcoma patients, CMB305 achieved a DCR of 61.9% and a median OS of 26.2 months, although no objective responses were observed (131, 132). The vaccine elicited NY-ESO-1-specific antibodies and T-cell responses in half of the patients. In a phase II study, combining CMB305 with atezolizumab yielded a median progression-free survival (PFS) of 2.6 months and OS of 18 months, with select patients demonstrating anti-NY-ESO-1 responses and improved outcomes (133).

## Cytokine-based therapy for sarcoma

Cytokines are soluble proteins mediating cellular interaction and immune response. Crucial cytokines like interleukins (ILs) and interferons (IFNs) modulate immune activity, and have been explored for their anti-tumor effects. IFNα was among the first agents used to treat HIV-related KS, showing tumor suppression in some patients (134). However, its clinical use is limited by low response and toxicity (135). While novel agents like liposomal doxorubicin and paclitaxel have supplanted IFNα as KS therapy, it may still have a role when combined with agents targeted angiogenic or HHV-8-encoded homologs (136).

IL-2 stimulates T and NK cells, promoting lymphocyte proliferation and activating lymphocytes into lymphokine-activated killer (LAK) cells, which can eradicate tumor cells independent of histocompatibility (137, 138). High-dose IL-2 has been approved for advanced melanoma but limited by severe toxicity (139). Small studies in sarcoma have shown IL-2, even conjunction with LAK cells or IFNs, offers limited antitumor effects (140–142). Nevertheless, IL-2-induced immune activation suggests potential efficacy for a subset of sarcoma.

IL-12, another potent anti-tumor cytokine, has shown promise in treating T-cell lymphoma and AIDS-related KS (143, 144). Like IL-2, the clinical application was limited by short half-life and toxicity. Recent studies on engineered IL-2 and IL-12 have demonstrated efficacy and safety in canine STS models by localizing effects and reducing systemic toxicity (145). Additionally, other cytokines like VEGFs, GM-CSFs, TGFs, and IFN-γ are under extensive study in clinical trials (146, 147).

## Conclusion and future direction

In conclusion, immunotherapy holds significant promise for advanced sarcomas, yet many sarcoma subtypes remain poorly responsive to ICIs due to their ‘cold’ immune microenvironment. In this regard, a recent review highlights the need for a more refined selection of patients based on immune biomarkers such as TLS and PD-L1 expression (148). TLS has garnered considerable interest as a predictive biomarker of the response to ICI therapies, or possibly chemotherapy (149). Some studies supported the presence of TLS in sarcoma associated with enhanced T− and B−cell responses, underscoring their central role in shaping the immune landscape (150, 151). Additionally, responders to immunotherapy often exhibit higher PD−L1 levels on both TAMs and T cells (65). Unfortunately, most clinical trials to date have not stratified patients by these biomarkers, potentially leading to disappointing results. Future trials should perform a better stratification of patients to optimize outcomes across these heterogeneous tumors.

Moreover, it is also relevant to mention that combining genomic profiling with immunotherapy may further help refine patient selection and improve clinical outcomes in sarcomas (152–154). Next−generation sequencing in STS can identify specific molecular pathways linked to chemosensitivity, as shown in MFS and UPS analyses (155). This combined approach promises to personalize treatment and improve outcomes in heterogeneous sarcoma populations.

## References

[1] van der Graaf WTA Orbach D Judson IR Ferrari A. Soft tissue sarcomas in adolescents and young adults: A comparison with their paediatric and adult counterparts. Lancet Oncol. (2017) 18:e166–e75. doi: 10.1016/s1470-2045(17)30099-2 28271871

[2] Demetri GD Antonescu CR Bjerkehagen B Bovée J Boye K Chacón M. Diagnosis and management of tropomyosin receptor kinase (Trk) fusion sarcomas: expert recommendations from the world sarcoma network. Ann Oncol. (2020) 31:1506–17. doi: 10.1016/j.annonc.2020.08.2232 PMC7985805 32891793

[3] Network CGAR. Comprehensive and integrated genomic characterization of adult soft tissue sarcomas. Cell. (2017) 171:950–65.e28. doi: 10.1016/j.cell.2017.10.014 29100075 PMC5693358

[4] Tap WD Wagner AJ Schöffski P Martin-Broto J Krarup-Hansen A Ganjoo KN. Effect of doxorubicin plus olaratumab vs doxorubicin plus placebo on survival in patients with advanced soft tissue sarcomas: the announce randomized clinical trial. Jama. (2020) 323:1266–76. doi: 10.1001/jama.2020.1707 PMC7139275 32259228

[5] Seddon B Strauss SJ Whelan J Leahy M Woll PJ Cowie F. Gemcitabine and docetaxel versus doxorubicin as first-line treatment in previously untreated advanced unresectable or metastatic soft-tissue sarcomas (Geddis): A randomised controlled phase 3 trial. Lancet Oncol. (2017) 18:1397–410. doi: 10.1016/s1470-2045(17)30622-8 PMC5622179 28882536

[6] Demetri GD von Mehren M Jones RL Hensley ML Schuetze SM Staddon A. Efficacy and safety of trabectedin or dacarbazine for metastatic liposarcoma or leiomyosarcoma after failure of conventional chemotherapy: results of a phase iii randomized multicenter clinical trial. J Clin Oncol. (2016) 34:786–93. doi: 10.1200/jco.2015.62.4734 PMC5070559 26371143

[7] Schöffski P Chawla S Maki RG Italiano A Gelderblom H Choy E. Eribulin versus dacarbazine in previously treated patients with advanced liposarcoma or leiomyosarcoma: A randomised, open-label, multicentre, phase 3 trial. Lancet. (2016) 387:1629–37. doi: 10.1016/s0140-6736(15)01283-0 26874885

[8] van der Graaf WT Blay JY Chawla SP Kim DW Bui-Nguyen B Casali PG. Pazopanib for metastatic soft-tissue sarcoma (Palette): A randomised, double-blind, placebo-controlled phase 3 trial. Lancet. (2012) 379:1879–86. doi: 10.1016/s0140-6736(12)60651-5 22595799

[9] Dalal S Shan KS Thaw Dar NN Hussein A Ergle A. Role of immunotherapy in sarcomas. Int J Mol Sci. (2024) 25:1266. doi: 10.3390/ijms25021266 38279265 PMC10816403

[10] Lee AQ Hao C Pan M Ganjoo KN Bui N. Use of histologic and immunologic factors in sarcoma to predict response rates to immunotherapy. J Clin Oncol. (2024) 42:11569. doi: 10.1200/JCO.2024.42.16_suppl.11569

[11] Varol C Mildner A Jung S. Macrophages: development and tissue specialization. Annu Rev Immunol. (2015) 33:643–75. doi: 10.1146/annurev-immunol-032414-112220 25861979

[12] D’Angelo SP Shoushtari AN Agaram NP Kuk D Qin LX Carvajal RD. Prevalence of tumor-infiltrating lymphocytes and pd-L1 expression in the soft tissue sarcoma microenvironment. Hum Pathol. (2015) 46:357–65. doi: 10.1016/j.humpath.2014.11.001 PMC5505649 25540867

[13] Nyström H Jönsson M Nilbert M Carneiro A. Immune-cell infiltration in high-grade soft tissue sarcomas; prognostic implications of tumor-associated macrophages and B-cells. Acta Oncol. (2023) 62:33–9. doi: 10.1080/0284186x.2023.2172688 36786033

[14] Dancsok AR Gao D Lee AF Steigen SE Blay JY Thomas DM. Tumor-associated macrophages and macrophage-related immune checkpoint expression in sarcomas. Oncoimmunology. (2020) 9:1747340. doi: 10.1080/2162402x.2020.1747340 32313727 PMC7153829

[15] Cersosimo F Lonardi S Bernardini G Telfer B Mandelli GE Santucci A. Tumor-associated macrophages in osteosarcoma: from mechanisms to therapy. Int J Mol Sci. (2020) 21:5207. doi: 10.3390/ijms21155207 32717819 PMC7432207

[16] Fujiwara T Healey J Ogura K Yoshida A Kondo H Hata T. Role of tumor-associated macrophages in sarcomas. Cancers (Basel). (2021) 13:1086. doi: 10.3390/cancers13051086 33802565 PMC7961818

[17] Zając AE Czarnecka AM Rutkowski P. The role of macrophages in sarcoma tumor microenvironment and treatment. Cancers (Basel). (2023) 15:5294. doi: 10.3390/cancers15215294 37958467 PMC10648209

[18] Umakoshi M Nakamura A Tsuchie H Li Z Kudo-Asabe Y Miyabe K. Macrophage numbers in the marginal area of sarcomas predict clinical prognosis. Sci Rep. (2023) 13:1290. doi: 10.1038/s41598-023-28024-1 36690825 PMC9870999

[19] Lee C-H Espinosa I Vrijaldenhoven S Subramanian S Montgomery KD Zhu S. Prognostic significance of macrophage infiltration in leiomyosarcomas. Clin Cancer Res. (2008) 14:1423–30. doi: 10.1158/1078-0432.CCR-07-1712%JClinicalCancerResearch 18316565

[20] Smolle MA Herbsthofer L Goda M Granegger B Brcic I Bergovec M. Influence of tumor-infiltrating immune cells on local control rate, distant metastasis, and survival in patients with soft tissue sarcoma. Oncoimmunology. (2021) 10:1896658. doi: 10.1080/2162402x.2021.1896658 33763294 PMC7954425

[21] Iseulys R Anne GB Corinne B Gonzague DBP Marie K Jean-Yves B. The immune landscape of chondrosarcoma reveals an immunosuppressive environment in the dedifferentiated subtypes and exposes csfr1+ Macrophages as a promising therapeutic target. J Bone Oncol. (2020) 20:100271. doi: 10.1016/j.jbo.2019.100271 31956474 PMC6961717

[22] Hemminger JA Iwenofu OH. Ny-eso-1 is a sensitive and specific immunohistochemical marker for myxoid and round cell liposarcomas among related mesenchymal myxoid neoplasms. Mod Pathol. (2013) 26:1204–10. doi: 10.1038/modpathol.2013.65 23599152

[23] Petitprez F de Reyniès A Keung EZ Chen TW Sun CM Calderaro J. B cells are associated with survival and immunotherapy response in sarcoma. Nature. (2020) 577:556–60. doi: 10.1038/s41586-019-1906-8 31942077

[24] Boxberg M Steiger K Lenze U Rechl H von Eisenhart-Rothe R Wörtler K. Pd-L1 and pd-1 and characterization of tumor-infiltrating lymphocytes in high grade sarcomas of soft tissue - prognostic implications and rationale for immunotherapy. Oncoimmunology. (2018) 7:e1389366. doi: 10.1080/2162402x.2017.1389366 29399389 PMC5790346

[25] Oike N Kawashima H Ogose A Hotta T Hatano H Ariizumi T. Prognostic impact of the tumor immune microenvironment in synovial sarcoma. Cancer Sci. (2018) 109:3043–54. doi: 10.1111/cas.13769 PMC6172059 30133055

[26] Tomassen T Weidema ME Hillebrandt-Roeffen MHS van der Horst C Desar IME Flucke UE. Analysis of pd-1, pd-L1, and T-cell infiltration in angiosarcoma pathogenetic subgroups. Immunol Res. (2022) 70:256–68. doi: 10.1007/s12026-021-09259-4 PMC8916989 35043369

[27] Klaver Y Rijnders M Oostvogels A Wijers R Smid M Grünhagen D. Differential quantities of immune checkpoint-expressing cd8 T cells in soft tissue sarcoma subtypes. J Immunother Cancer. (2020) 8:e000271. doi: 10.1136/jitc-2019-000271 32792357 PMC7430493

[28] Keung EZ Burgess M Salazar R Parra ER Rodrigues-Canales J Bolejack V. Correlative analyses of the sarc028 trial reveal an association between sarcoma-associated immune infiltrate and response to pembrolizumab. Clin Cancer Res. (2020) 26:1258–66. doi: 10.1158/1078-0432.Ccr-19-1824 PMC7731262 31900276

[29] Anzar I Malone B Samarakoon P Vardaxis I Simovski B Fontenelle H. The interplay between neoantigens and immune cells in sarcomas treated with checkpoint inhibition. Front Immunol. (2023) 14:1226445. doi: 10.3389/fimmu.2023.1226445 37799721 PMC10548483

[30] D’Angelo SP Richards AL Conley AP Woo HJ Dickson MA Gounder M. Pilot study of bempegaldesleukin in combination with nivolumab in patients with metastatic sarcoma. Nat Commun. (2022) 13:3477. doi: 10.1038/s41467-022-30874-8 35710741 PMC9203519

[31] Que Y Xiao W Guan YX Liang Y Yan SM Chen HY. Pd-L1 expression is associated with foxp3+ Regulatory T-cell infiltration of soft tissue sarcoma and poor patient prognosis. J Cancer. (2017) 8:2018–25. doi: 10.7150/jca.18683 PMC5559963 28819402

[32] Smolle MA Herbsthofer L Granegger B Goda M Brcic I Bergovec M. T-regulatory cells predict clinical outcome in soft tissue sarcoma patients: A clinico-pathological study. Br J Cancer. (2021) 125:717–24. doi: 10.1038/s41416-021-01456-0 PMC8405702 34127811

[33] Maleki Vareki S. High and low mutational burden tumors versus immunologically hot and cold tumors and response to immune checkpoint inhibitors. J Immunother Cancer. (2018) 6:157. doi: 10.1186/s40425-018-0479-7 30587233 PMC6307306

[34] Marabelle A Fakih M Lopez J Shah M Shapira-Frommer R Nakagawa K. Association of tumour mutational burden with outcomes in patients with advanced solid tumours treated with pembrolizumab: prospective biomarker analysis of the multicohort, open-label, phase 2 keynote-158 study. Lancet Oncol. (2020) 21:1353–65. doi: 10.1016/s1470-2045(20)30445-9 32919526

[35] Wood GE Meyer C Petitprez F D’Angelo SP. Immunotherapy in sarcoma: current data and promising strategies. Am Soc Clin Oncol Educ Book. (2024) 44:e432234. doi: 10.1200/edbk_432234 38781557

[36] Trabucco SE Ali SM Sokol E Schrock AB Albacker LA Chung J. Frequency of genomic biomarkers of response to immunotherapy in sarcoma. J Clin Oncol. (2018) 36:11579. doi: 10.1200/JCO.2018.36.15_suppl.11579

[37] Denu RA Moyers JT Gouda MA Conley AP Lazar AJ Subbiah V. The landscape of alterations from 1407 ultra-rare sarcomas from the aacr genie database: clinical implications. Clin Cancer Res. (2023) 29:4669–78. doi: 10.1158/1078-0432.Ccr-23-0876 PMC11874058 37643131

[38] Espejo-Freire AP Elliott A Rosenberg A Costa PA Barreto-Coelho P Jonczak E. Genomic landscape of angiosarcoma: A targeted and immunotherapy biomarker analysis. Cancers (Basel). (2021) 13:4816. doi: 10.3390/cancers13194816 34638300 PMC8507700

[39] Chalmers ZR Connelly CF Fabrizio D Gay L Ali SM Ennis R. Analysis of 100,000 human cancer genomes reveals the landscape of tumor mutational burden. Genome Med. (2017) 9:34. doi: 10.1186/s13073-017-0424-2 28420421 PMC5395719

[40] He M Abro B Kaushal M Chen L Chen T Gondim M. Tumor mutation burden and checkpoint immunotherapy markers in primary and metastatic synovial sarcoma. Hum Pathol. (2020) 100:15–23. doi: 10.1016/j.humpath.2020.04.007 32387103

[41] Wagner MJ Othus M Patel SP Ryan C Sangal A Powers B. Multicenter phase ii trial (Swog S1609, cohort 51) of ipilimumab and nivolumab in metastatic or unresectable angiosarcoma: A substudy of dual anti-ctla-4 and anti-pd-1 blockade in rare tumors (Dart). J Immunother Cancer. (2021) 9:e002990. doi: 10.1136/jitc-2021-002990 34380663 PMC8330584

[42] Painter CA Jain E Tomson BN Dunphy M Stoddard RE Thomas BS. The angiosarcoma project: enabling genomic and clinical discoveries in a rare cancer through patient-partnered research. Nat Med. (2020) 26:181–7. doi: 10.1038/s41591-019-0749-z 32042194

[43] Yan L Wang Z Cui C Guan X Dong B Zhao M. Comprehensive immune characterization and T-cell receptor repertoire heterogeneity of retroperitoneal liposarcoma. Cancer Sci. (2019) 110:3038–48. doi: 10.1111/cas.14161 PMC6778648 31385405

[44] Wedekind MF Haworth KB Arnold M Stanek JR Lee D Cripe TP. Immune profiles of desmoplastic small round cell tumor and synovial sarcoma suggest different immunotherapeutic susceptibility upfront compared to relapse specimens. Pediatr Blood Cancer. (2018) 65:e27313. doi: 10.1002/pbc.27313 30015384

[45] Patel KR Martinez A Stahl JM Logan SJ Perricone AJ Ferris MJ. Increase in pd-L1 expression after pre-operative radiotherapy for soft tissue sarcoma. Oncoimmunology. (2018) 7:e1442168. doi: 10.1080/2162402x.2018.1442168 29900051 PMC5993497

[46] Fridman WH Meylan M Petitprez F Sun CM Italiano A Sautès-Fridman C. B cells and tertiary lymphoid structures as determinants of tumour immune contexture and clinical outcome. Nat Rev Clin Oncol. (2022) 19:441–57. doi: 10.1038/s41571-022-00619-z 35365796

[47] Nielsen JS Nelson BH. Tumor-infiltrating B cells and T cells: working together to promote patient survival. Oncoimmunology. (2012) 1:1623–5. doi: 10.4161/onci.21650 PMC3525624 23264915

[48] Bruno TC Ebner PJ Moore BL Squalls OG Waugh KA Eruslanov EB. Antigen-presenting intratumoral B cells affect cd4(+) til phenotypes in non-small cell lung cancer patients. Cancer Immunol Res. (2017) 5:898–907. doi: 10.1158/2326-6066.Cir-17-0075 28848053 PMC5788174

[49] Wang WW Yuan XL Chen H Xie GH Ma YH Zheng YX. Cd19+Cd24hicd38hibregs involved in downregulate helper T cells and upregulate regulatory T cells in gastric cancer. Oncotarget. (2015) 6:33486–99. doi: 10.18632/oncotarget.5588 PMC4741780 26378021

[50] Shao Y Lo CM Ling CC Liu XB Ng KT Chu AC. Regulatory B cells accelerate hepatocellular carcinoma progression via cd40/cd154 signaling pathway. Cancer Lett. (2014) 355:264–72. doi: 10.1016/j.canlet.2014.09.026 25301451

[51] Zhang Y Xu M Ren Y Ba Y Liu S Zuo A. Tertiary lymphoid structural heterogeneity determines tumour immunity and prospects for clinical application. Mol Cancer. (2024) 23:75. doi: 10.1186/s12943-024-01980-6 38582847 PMC10998345

[52] Italiano A Bessede A Pulido M Bompas E Piperno-Neumann S Chevreau C. Pembrolizumab in soft-tissue sarcomas with tertiary lymphoid structures: A phase 2 pembrosarc trial cohort. Nat Med. (2022) 28:1199–206. doi: 10.1038/s41591-022-01821-3 35618839

[53] Meylan M Petitprez F Becht E Bougoüin A Pupier G Calvez A. Tertiary lymphoid structures generate and propagate anti-tumor antibody-producing plasma cells in renal cell cancer. Immunity. (2022) 55:527–41.e5. doi: 10.1016/j.immuni.2022.02.001 35231421

[54] Patil NS Nabet BY Müller S Koeppen H Zou W Giltnane J. Intratumoral plasma cells predict outcomes to pd-L1 blockade in non-small cell lung cancer. Cancer Cell. (2022) 40:289–300.e4. doi: 10.1016/j.ccell.2022.02.002 35216676

[55] Cho HJ Yun KH Shin SJ Lee YH Kim SH Baek W. Durvalumab plus pazopanib combination in patients with advanced soft tissue sarcomas: A phase ii trial. Nat Commun. (2024) 15:685. doi: 10.1038/s41467-024-44875-2 38263321 PMC10806253

[56] Munn DH Sharma MD Baban B Harding HP Zhang Y Ron D. Gcn2 kinase in T cells mediates proliferative arrest and anergy induction in response to indoleamine 2,3-dioxygenase. Immunity. (2005) 22:633–42. doi: 10.1016/j.immuni.2005.03.013 15894280

[57] Sharma MD Baban B Chandler P Hou DY Singh N Yagita H. Plasmacytoid dendritic cells from mouse tumor-draining lymph nodes directly activate mature tregs via indoleamine 2,3-dioxygenase. J Clin Invest. (2007) 117:2570–82. doi: 10.1172/jci31911 PMC1940240 17710230

[58] Toulmonde M Adam J Bessede A Ranchère-Vince D Velasco V Brouste V. Integrative assessment of expression and prognostic value of pdl1, ido, and kynurenine in 371 primary soft tissue sarcomas with genomic complexity. J Clin Oncol. (2016) 34:11008–. doi: 10.1200/JCO.2016.34.15_suppl.11008

[59] Toulmonde M Penel N Adam J Chevreau C Blay JY Le Cesne A. Use of pd-1 targeting, macrophage infiltration, and ido pathway activation in sarcomas: A phase 2 clinical trial. JAMA Oncol. (2018) 4:93–7. doi: 10.1001/jamaoncol.2017.1617 PMC5833654 28662235

[60] Long GV Dummer R Hamid O Gajewski TF Caglevic C Dalle S. Epacadostat plus pembrolizumab versus placebo plus pembrolizumab in patients with unresectable or metastatic melanoma (Echo-301/keynote-252): A phase 3, randomised, double-blind study. Lancet Oncol. (2019) 20:1083–97. doi: 10.1016/s1470-2045(19)30274-8 31221619

[61] Kelly CM Qin LX Whiting KA Richards AL Avutu V Chan JE. A phase ii study of epacadostat and pembrolizumab in patients with advanced sarcoma. Clin Cancer Res. (2023) 29:2043–51. doi: 10.1158/1078-0432.Ccr-22-3911 PMC10752758 36971773

[62] Ben-Ami E Barysauskas CM Solomon S Tahlil K Malley R Hohos M. Immunotherapy with single agent nivolumab for advanced leiomyosarcoma of the uterus: results of a phase 2 study. Cancer. (2017) 123:3285–90. doi: 10.1002/cncr.30738 PMC5762200 28440953

[63] Maki RG Jungbluth AA Gnjatic S Schwartz GK D’Adamo DR Keohan ML. A pilot study of anti-ctla4 antibody ipilimumab in patients with synovial sarcoma. Sarcoma. (2013) 2013:168145. doi: 10.1155/2013/168145 23554566 PMC3608267

[64] Tawbi HA Burgess M Bolejack V Van Tine BA Schuetze SM Hu J. Pembrolizumab in advanced soft-tissue sarcoma and bone sarcoma (Sarc028): A multicentre, two-cohort, single-arm, open-label, phase 2 trial. Lancet Oncol. (2017) 18:1493–501. doi: 10.1016/s1470-2045(17)30624-1 PMC7939029 28988646

[65] Blay JY Chevret S Le Cesne A Brahmi M Penel N Cousin S. Pembrolizumab in patients with rare and ultra-rare sarcomas (Acsé Pembrolizumab): analysis of a subgroup from a non-randomised, open-label, phase 2, basket trial. Lancet Oncol. (2023) 24:892–902. doi: 10.1016/s1470-2045(23)00282-6 37429302

[66] Italiano A Bellera C D’Angelo S. Pd1/pd-L1 targeting in advanced soft-tissue sarcomas: A pooled analysis of phase ii trials. J Hematol Oncol. (2020) 13:55. doi: 10.1186/s13045-020-00891-5 32430039 PMC7236113

[67] Postow MA Chesney J Pavlick AC Robert C Grossmann K McDermott D. Nivolumab and ipilimumab versus ipilimumab in untreated melanoma. N Engl J Med. (2015) 372:2006–17. doi: 10.1056/NEJMoa1414428 PMC5744258 25891304

[68] D’Angelo SP Mahoney MR Van Tine BA Atkins J Milhem MM Jahagirdar BN. Nivolumab with or without ipilimumab treatment for metastatic sarcoma (Alliance A091401): two open-label, non-comparative, randomised, phase 2 trials. Lancet Oncol. (2018) 19:416–26. doi: 10.1016/s1470-2045(18)30006-8 PMC6126546 29370992

[69] Roland CL Nassif Haddad EF Keung EZ Wang WL Lazar AJ Lin H. A randomized, non-comparative phase 2 study of neoadjuvant immune-checkpoint blockade in retroperitoneal dedifferentiated liposarcoma and extremity/truncal undifferentiated pleomorphic sarcoma. Nat Cancer. (2024) 5:625–41. doi: 10.1038/s43018-024-00726-z PMC12955605 38351182

[70] Chen AP Sharon E O’Sullivan-Coyne G Moore N Foster JC Hu JS. Atezolizumab for advanced alveolar soft part sarcoma. N Engl J Med. (2023) 389:911–21. doi: 10.1056/NEJMoa2303383 PMC10729808 37672694

[71] Pollack SM Redman MW Baker KK Wagner MJ Schroeder BA Loggers ET. Assessment of doxorubicin and pembrolizumab in patients with advanced anthracycline-naive sarcoma: A phase 1/2 nonrandomized clinical trial. JAMA Oncol. (2020) 6:1778–82. doi: 10.1001/jamaoncol.2020.3689 PMC7489365 32910151

[72] Livingston MB Jagosky MH Robinson MM Ahrens WA Benbow JH Farhangfar CJ. Phase ii study of pembrolizumab in combination with doxorubicin in metastatic and unresectable soft-tissue sarcoma. Clin Cancer Res. (2021) 27:6424–31. doi: 10.1158/1078-0432.Ccr-21-2001 34475102

[73] Reichardt P Andreou D Flörcken A Groß T Richter S Kessler T. Efficacy and safety of nivolumab and trabectedin in pretreated patients with advanced soft tissue sarcomas (Sts): results of a phase ii trial of the german interdisciplinary sarcoma group (Gisg-15, nitrasarc). J Clin Oncol. (2023) 41:11500. doi: 10.1200/JCO.2023.41.16_suppl.11500

[74] Gordon EM Chawla SP Tellez WA Younesi E Thomas S Chua-Alcala VS. Saint: A phase I/expanded phase ii study using safe amounts of ipilimumab, nivolumab and trabectedin as first-line treatment of advanced soft tissue sarcoma. Cancers (Basel). (2023) 15:906. doi: 10.3390/cancers15030906 36765863 PMC9913367

[75] Haddox CL Nathenson MJ Mazzola E Lin JR Baginska J Nau A. Phase ii study of eribulin plus pembrolizumab in metastatic soft-tissue sarcomas: clinical outcomes and biological correlates. Clin Cancer Res. (2024) 30:1281–92. doi: 10.1158/1078-0432.Ccr-23-2250 PMC10982640 38236580

[76] Wilky BA Trucco MM Subhawong TK Florou V Park W Kwon D. Axitinib plus pembrolizumab in patients with advanced sarcomas including alveolar soft-part sarcoma: A single-centre, single-arm, phase 2 trial. Lancet Oncol. (2019) 20:837–48. doi: 10.1016/s1470-2045(19)30153-6 31078463

[77] Martin-Broto J Hindi N Grignani G Martinez-Trufero J Redondo A Valverde C. Nivolumab and sunitinib combination in advanced soft tissue sarcomas: A multicenter, single-arm, phase ib/ii trial. J Immunother Cancer. (2020) 8:e001561. doi: 10.1136/jitc-2020-001561 33203665 PMC7674086

[78] Liu J Gao T Tan Z Li S Xu J Bai C. Phase ii study of tqb2450, a novel pd-L1 antibody, in combination with anlotinib in patients with locally advanced or metastatic soft tissue sarcoma. Clin Cancer Res. (2022) 28:3473–9. doi: 10.1158/1078-0432.Ccr-22-0871 PMC9662895 35675031

[79] Zer A Icht O Yosef L Avram D Jacobi O Fenig E. Phase ii single-arm study of nivolumab and ipilimumab (Nivo/ipi) in previously treated classical kaposi sarcoma (Cks). Ann Oncol. (2022) 33:720–7. doi: 10.1016/j.annonc.2022.03.012 35339649

[80] Yang J Dong L Yang S Han X Han Y Jiang S. Safety and clinical efficacy of toripalimab, a pd-1 mab, in patients with advanced or recurrent Malignancies in a phase I study. Eur J Cancer. (2020) 130:182–92. doi: 10.1016/j.ejca.2020.01.028 32224416

[81] Peraza DA Povo-Retana A Mojena M García-Redondo AB Avilés P Boscá L. Trabectedin modulates macrophage polarization in the tumor-microenvironment. Role of K(V)1.3 and K(V)1.5 channels. BioMed Pharmacother. (2023) 161:114548. doi: 10.1016/j.biopha.2023.114548 36940615

[82] Sun CM Toulmonde M Spalato-Ceruso M Peyraud F Bessede A Kind M. Impact of metronomic trabectedin combined with low-dose cyclophosphamide on sarcoma microenvironment and correlation with clinical outcome: results from the tarmic study. Mol Cancer. (2024) 23:37. doi: 10.1186/s12943-024-01942-y 38374062 PMC10875852

[83] Gordon EM Chawla SP Omelchenko N Jeffrey S Agarwal AD Kumar V. Seven year update on soc-1702: A phase 2 study using trabectedin (T) in combination with ipilimumab (I), nivolumab (N) and trabectedin (T) in previously untreated patients with advanced soft tissue sarcoma. J Clin Oncol. (2024) 42:11577. doi: 10.1200/JCO.2024.42.16_suppl.11577

[84] Lee WS Yang H Chon HJ Kim C. Combination of anti-angiogenic therapy and immune checkpoint blockade normalizes vascular-immune crosstalk to potentiate cancer immunity. Exp Mol Med. (2020) 52:1475–85. doi: 10.1038/s12276-020-00500-y PMC8080646 32913278

[85] Ocadlikova D Lecciso M Broto JM Scotlandi K Cavo M Curti A. Sunitinib exerts *in vitro* immunomodulatory activity on sarcomas via dendritic cells and synergizes with pd-1 blockade. Front Immunol. (2021) 12:577766. doi: 10.3389/fimmu.2021.577766 33717062 PMC7952316

[86] Muñiz NH Trufero JM Grignani G Garcia AS Strauss S Stacchiotti S. 1922p immunosarc ii master trial: phase ii of sunitinib and nivolumab in vascular sarcomas cohort - a geis, isg and ucl study. Ann Oncol. (2023) 34:S1034. doi: 10.1016/j.annonc.2023.09.1151

[87] Strauss SJ Hindi N Palmerini E Martínez-Trufero J Lopez-Pousa A Carrasco-Garcia I. Immunosarc ii master trial (Phase ii of sunitinib and nivolumab): results from the dedifferentiated chondrosarcoma (Ddcs) cohort—a geis, isg and ucl study. J Clin Oncol. (2024) 42:11506. doi: 10.1200/JCO.2024.42.16_suppl.11506

[88] Chi Y Fang Z Hong X Yao Y Sun P Wang G. Safety and efficacy of anlotinib, a multikinase angiogenesis inhibitor, in patients with refractory metastatic soft-tissue sarcoma. Clin Cancer Res. (2018) 24:5233–8. doi: 10.1158/1078-0432.Ccr-17-3766 29895706

[89] Tan Z Wu Y Fan Z Liu J Gao T Bai C. A phase ii study of anlotinib and an anti-pdl1 antibody in patients with alveolar soft part sarcoma: results of expansion cohorts. J Clin Oncol. (2024) 42:11515. doi: 10.1200/JCO.2024.42.16_suppl.11515

[90] Liu J Fan Z Bai C Li S Xue R Gao T. Real-world experience with pembrolizumab in patients with advanced soft tissue sarcoma. Ann Transl Med. (2021) 9:339. doi: 10.21037/atm-21-49 33708966 PMC7944271

[91] Nasr LF Zoghbi M Lazcano R Nakazawa M Bishop AJ Farooqi A. High-grade pleomorphic sarcomas treated with immune checkpoint blockade: the md anderson cancer center experience. Cancers (Basel). (2024) 16:1763. doi: 10.3390/cancers16091763 38730715 PMC11083765

[92] Li S Sun Q Bai R Wang Y Wang H Chen H. Real-world efficacy, safety data and predictive clinical parameters for treatment outcomes in advanced soft tissue sarcoma treated with combined immunotherapy and antiangiogenic therapy. BMC Cancer. (2024) 24:1028. doi: 10.1186/s12885-024-12810-9 39164643 PMC11337792

[93] Liu Z Xu J Liu M Hu W Xu N Zhu D. Efficacy and safety of angiogenesis inhibitors plus immune checkpoint inhibitors in advanced soft tissue sarcoma: A real-world, single-center study. Sci Rep. (2023) 13:3385. doi: 10.1038/s41598-023-30412-6 36854710 PMC9974953

[94] D’Angelo SP Furness AJS Thistlethwaite F Burgess MA Riedel RF Haanen J. Lete-cel in patients with synovial sarcoma or myxoid/round cell liposarcoma: planned interim analysis of the pivotal ignyte-eso trial. J Clin Oncol. (2024) 42:2500–. doi: 10.1200/JCO.2024.42.16_suppl.2500

[95] Pan Q Weng D Liu J Han Z Ou Y Xu B. Phase 1 clinical trial to assess safety and efficacy of ny-eso-1-specific tcr T cells in hla-a∗02:01 patients with advanced soft tissue sarcoma. Cell Rep Med. (2023) 4:101133. doi: 10.1016/j.xcrm.2023.101133 37586317 PMC10439245

[96] D’Angelo SP Araujo DM Abdul Razak AR Agulnik M Attia S Blay JY. Afamitresgene autoleucel for advanced synovial sarcoma and myxoid round cell liposarcoma (Spearhead-1): an international, open-label, phase 2 trial. Lancet. (2024) 403:1460–71. doi: 10.1016/s0140-6736(24)00319-2 PMC11419333 38554725

[97] Sidaway P. Afami-cel provides a novel treatment option for rare sarcoma subtypes. Nat Rev Clin Oncol. (2024) 21:401. doi: 10.1038/s41571-024-00894-y 38637649

[98] Lynch MM Obeidin F Alexiev BA Chen EY Viveiros P Schroeder B. (2023). B7h3 expression in sarcoma. J Clin Oncol. (2023) 41:e23517. doi: 10.1200/JCO.2023.41.16_suppl.e23517

[99] de Bono JS Helissey C Fizazi K Maroto Rey JP Roubaud G Antonarakis ES. 1654p tamarack: randomized phase ii trial of the B7-H3 targeting antibody drug conjugate (Adc) vobramitamab duocarmazine (Vobra duo) in metastatic castration-resistant prostate cancer (Mcrpc). Ann Oncol. (2024) 35:S996–S7. doi: 10.1016/j.annonc.2024.08.1735

[100] Vitanza NA Wilson AL Huang W Seidel K Brown C Gustafson JA. Intraventricular B7-H3 car T cells for diffuse intrinsic pontine glioma: preliminary first-in-human bioactivity and safety. Cancer Discov. (2023) 13:114–31. doi: 10.1158/2159-8290.Cd-22-0750 PMC9827115 36259971

[101] Pinto NR Albert CM Taylor M Wilson A Rawlings-Rhea S Huang W. Strive-02: A first-in-human phase 1 trial of systemic B7h3 car T cells for children and young adults with relapsed/refractory solid tumors. J Clin Oncol. (2022) 40:10011–10011. doi: 10.1200/JCO.2022.40.16_suppl.10011

[102] Pinto N Albert CM Taylor MR Ullom HB Wilson AL Huang W. Strive-02: A first-in-human phase I study of systemically administered B7-H3 chimeric antigen receptor T cells for patients with relapsed/refractory solid tumors. J Clin Oncol. (2024) 42:4163–72. doi: 10.1200/jco.23.02229 39255444

[103] Choe M Taylor M Wendler J Narayanaswamy P Rawlings-Rhea S Seidel K. Strive-02 arm C: phase 1 study of concurrent pd-1 inhibition with B7-H3 car T cell immunotherapy for recurrent/refractory pediatric solid tumors. J Clin Oncol. (2024) 42:e14626. doi: 10.1200/JCO.2024.42.16_suppl.e14626

[104] Pinto NR Albert CM Taylor M Wilson A Rawlings-Rhea S Huang W. Effect of bispecific B7h3 X cd19 car T cells on host cd19 expression and car T cell engraftment. J Clin Oncol. (2023) 41:10043–. doi: 10.1200/JCO.2023.41.16_suppl.10043

[105] Talbot LJ Chabot A Ross AB Beckett A Nguyen P Fleming A. Redirecting B7-H3.Car T cells to chemokines expressed in osteosarcoma enhances homing and antitumor activity in preclinical models. Clin Cancer Res. (2024) 30:4434–49. doi: 10.1158/1078-0432.Ccr-23-3298 PMC11443211 39101835

[106] Hegde M Navai S DeRenzo C Joseph SK Sanber K Wu M. Autologous her2-specific car T cells after lymphodepletion for advanced sarcoma: A phase 1 trial. Nat Cancer. (2024) 5:880–94. doi: 10.1038/s43018-024-00749-6 PMC11588040 38658775

[107] Del Bufalo F De Angelis B Caruana I Del Baldo G De Ioris MA Serra A. Gd2-cart01 for relapsed or refractory high-risk neuroblastoma. N Engl J Med. (2023) 388:1284–95. doi: 10.1056/NEJMoa2210859 37018492

[108] Majzner RG Ramakrishna S Yeom KW Patel S Chinnasamy H Schultz LM. Gd2-car T cell therapy for H3k27m-mutated diffuse midline gliomas. Nature. (2022) 603:934–41. doi: 10.1038/s41586-022-04489-4 PMC8967714 35130560

[109] Kaczanowska S Murty T Alimadadi A Contreras CF Duault C Subrahmanyam PB. Immune determinants of car-T cell expansion in solid tumor patients receiving gd2 car-T cell therapy. Cancer Cell. (2024) 42:35–51.e8. doi: 10.1016/j.ccell.2023.11.011 38134936 PMC10947809

[110] Uslu U June CH. Beyond the blood: expanding car T cell therapy to solid tumors. Nat Biotechnol. (2024) 43:506–15. doi: 10.1038/s41587-024-02446-2 39533105

[111] Rosenberg SA Yang JC Sherry RM Kammula US Hughes MS Phan GQ. Durable complete responses in heavily pretreated patients with metastatic melanoma using T-cell transfer immunotherapy. Clin Cancer Res. (2011) 17:4550–7. doi: 10.1158/1078-0432.Ccr-11-0116 PMC3131487 21498393

[112] Andersen R Donia M Ellebaek E Borch TH Kongsted P Iversen TZ. Long-lasting complete responses in patients with metastatic melanoma after adoptive cell therapy with tumor-infiltrating lymphocytes and an attenuated il2 regimen. Clin Cancer Res. (2016) 22:3734–45. doi: 10.1158/1078-0432.Ccr-15-1879 27006492

[113] Rohaan MW Borch TH van den Berg JH Met Ö Kessels R Geukes Foppen MH. Tumor-infiltrating lymphocyte therapy or ipilimumab in advanced melanoma. N Engl J Med. (2022) 387:2113–25. doi: 10.1056/NEJMoa2210233 36477031

[114] Nielsen M Monberg T Sundvold V Albieri B Hovgaard D Petersen MM. Ltx-315 and adoptive cell therapy using tumor-infiltrating lymphocytes generate tumor specific T cells in patients with metastatic soft tissue sarcoma. Oncoimmunology. (2024) 13:2290900. doi: 10.1080/2162402x.2023.2290900 38125722 PMC10732595

[115] Li XQ Yamazaki T He T Alam MM Liu J Trivett AL. Ltx-315 triggers anticancer immunity by inducing myd88-dependent maturation of dendritic cells. Front Immunol. (2024) 15:1332922. doi: 10.3389/fimmu.2024.1332922 38545099 PMC10967226

[116] Toulmonde M Cousin S Kind M Guegan JP Bessede A Le Loarer F. Randomized phase 2 trial of intravenous oncolytic virus jx-594 combined with low-dose cyclophosphamide in patients with advanced soft-tissue sarcoma. J Hematol Oncol. (2022) 15:149. doi: 10.1186/s13045-022-01370-9 36271420 PMC9585864

[117] Toulmonde M Guegan JP Spalato-Ceruso M Peyraud F Kind M Vanhersecke L. Reshaping the tumor microenvironment of cold soft-tissue sarcomas with oncolytic viral therapy: A phase 2 trial of intratumoral jx-594 combined with avelumab and low-dose cyclophosphamide. Mol Cancer. (2024) 23:38. doi: 10.1186/s12943-024-01946-8 38378555 PMC10877825

[118] Monga V Miller BJ Tanas M Boukhar S Allen B Anderson C. Intratumoral talimogene laherparepvec injection with concurrent preoperative radiation in patients with locally advanced soft-tissue sarcoma of the trunk and extremities: phase ib/ii trial. J Immunother Cancer. (2021) 9:e003119. doi: 10.1136/jitc-2021-003119 34330766 PMC8327848

[119] Kelly CM Antonescu CR Bowler T Munhoz R Chi P Dickson MA. Objective response rate among patients with locally advanced or metastatic sarcoma treated with talimogene laherparepvec in combination with pembrolizumab: A phase 2 clinical trial. JAMA Oncol. (2020) 6:402–8. doi: 10.1001/jamaoncol.2019.6152 PMC6990941 31971541

[120] Kelly CM Avutu V Chi P Dickson MA Gounder MM Keohan ML. A phase ii study of talimogene laherparepvec (T-vec) and pembrolizumab in patients with advanced sarcoma: results of expansion cohorts. J Clin Oncol. (2023) 41:11570. doi: 10.1200/JCO.2023.41.16_suppl.11570

[121] Tan Z Liu J Gao T Ding S Han L Luo S. A phase ii study of an oncolytic herpes simplex virus 2 and an anti-pd-1 antibody in patients with advanced sarcoma. J Clin Oncol. (2024) 42:11571. doi: 10.1200/JCO.2024.42.16_suppl.11571

[122] Tang T Huang X Zhang G Liang T. Oncolytic immunotherapy: multiple mechanisms of oncolytic peptides to confer anticancer immunity. J Immunother Cancer. (2022) 10:e005065. doi: 10.1136/jitc-2022-005065 35851309 PMC9295653

[123] Takahashi R Ishibashi Y Hiraoka K Matsueda S Kawano K Kawahara A. Phase ii study of personalized peptide vaccination for refractory bone and soft tissue sarcoma patients. Cancer Sci. (2013) 104:1285–94. doi: 10.1111/cas.12226 PMC7656559 23829867

[124] Muraoka D Harada N Hayashi T Tahara Y Momose F Sawada S. Nanogel-based immunologically stealth vaccine targets macrophages in the medulla of lymph node and induces potent antitumor immunity. ACS Nano. (2014) 8:9209–18. doi: 10.1021/nn502975r 25180962

[125] Ishihara M Nishida Y Kitano S Kawai A Muraoka D Momose F. A phase 1 trial of ny-eso-1-specific tcr-engineered T-cell therapy combined with a lymph node-targeting nanoparticulate peptide vaccine for the treatment of advanced soft tissue sarcoma. Int J Cancer. (2023) 152:2554–66. doi: 10.1002/ijc.34453 36727538

[126] Najafi S Mortezaee K. Advances in dendritic cell vaccination therapy of cancer. Biomedicine Pharmacotherapy. (2023) 164:114954. doi: 10.1016/j.biopha.2023.114954 37257227

[127] Miwa S Nishida H Tanzawa Y Takeuchi A Hayashi K Yamamoto N. Phase 1/2 study of immunotherapy with dendritic cells pulsed with autologous tumor lysate in patients with refractory bone and soft tissue sarcoma. Cancer. (2017) 123:1576–84. doi: 10.1002/cncr.30606 28241093

[128] Fedorova L Mudry P Pilatova K Selingerova I Merhautova J Rehak Z. Assessment of immune response following dendritic cell-based immunotherapy in pediatric patients with relapsing sarcoma. Front Oncol. (2019) 9:1169. doi: 10.3389/fonc.2019.01169 31799177 PMC6868036

[129] Pollack SM Lu H Gnjatic S Somaiah N O’Malley RB Jones RL. First-in-human treatment with a dendritic cell-targeting lentiviral vector-expressing ny-eso-1, lv305, induces deep, durable response in refractory metastatic synovial sarcoma patient. J Immunother. (2017) 40:302–6. doi: 10.1097/cji.0000000000000183 PMC5733794 28891906

[130] Akahori Y Wang L Yoneyama M Seo N Okumura S Miyahara Y. Antitumor activity of car-T cells targeting the intracellular oncoprotein wt1 can be enhanced by vaccination. Blood. (2018) 132:1134–45. doi: 10.1182/blood-2017-08-802926 PMC6148344 30045840

[131] Somaiah N Chawla SP Block MS Morris JC Do K Kim JW. A phase 1b study evaluating the safety, tolerability, and immunogenicity of cmb305, a lentiviral-based prime-boost vaccine regimen, in patients with locally advanced, relapsed, or metastatic cancer expressing ny-eso-1. Oncoimmunology. (2020) 9:1847846. doi: 10.1080/2162402x.2020.1847846 33312760 PMC7714520

[132] Pollack SM. The potential of the cmb305 vaccine regimen to target ny-eso-1 and improve outcomes for synovial sarcoma and myxoid/round cell liposarcoma patients. Expert Rev Vaccines. (2018) 17:107–14. doi: 10.1080/14760584.2018.1419068 PMC6521962 29280411

[133] Chawla SP Van Tine BA Pollack SM Ganjoo KN Elias AD Riedel RF. Phase ii randomized study of cmb305 and atezolizumab compared with atezolizumab alone in soft-tissue sarcomas expressing ny-eso-1. J Clin Oncol. (2022) 40:1291–300. doi: 10.1200/jco.20.03452 34260265

[134] Groopman JE Gottlieb MS Goodman J Mitsuyasu RT Conant MA Prince H. Recombinant alpha-2 interferon therapy for kaposi’s sarcoma associated with the acquired immunodeficiency syndrome. Ann Intern Med. (1984) 100:671–6. doi: 10.7326/0003-4819-100-5-671 6712031

[135] Berraondo P Sanmamed MF Ochoa MC Etxeberria I Aznar MA Pérez-Gracia JL. Cytokines in clinical cancer immunotherapy. Br J Cancer. (2019) 120:6–15. doi: 10.1038/s41416-018-0328-y 30413827 PMC6325155

[136] Krown SE. Aids-associated kaposi’s sarcoma: is there still a role for interferon alfa? Cytokine Growth Factor Rev. (2007) 18:395–402. doi: 10.1016/j.cytogfr.2007.06.005 17656146 PMC2041795

[137] Fagan EA Eddleston AL. Immunotherapy for cancer: the use of lymphokine activated killer (Lak) cells. Gut. (1987) 28:113–6. doi: 10.1136/gut.28.2.113 PMC1432985 3549471

[138] Schwinger W Klass V Benesch M Lackner H Dornbusch HJ Sovinz P. Feasibility of high-dose interleukin-2 in heavily pretreated pediatric cancer patients. Ann Oncol. (2005) 16:1199–206. doi: 10.1093/annonc/mdi226 15849223

[139] Atkins MB Lotze MT Dutcher JP Fisher RI Weiss G Margolin K. High-dose recombinant interleukin 2 therapy for patients with metastatic melanoma: analysis of 270 patients treated between 1985 and 1993. J Clin Oncol. (1999) 17:2105–16. doi: 10.1200/jco.1999.17.7.2105 10561265

[140] Rosenberg SA Lotze MT Yang JC Aebersold PM Linehan WM Seipp CA. Experience with the use of high-dose interleukin-2 in the treatment of 652 cancer patients. Ann Surg. (1989) 210:474–84. doi: 10.1097/00000658-198910000-00008 PMC1357927 2679456

[141] Bauer M Reaman GH Hank JA Cairo MS Anderson P Blazar BR. A phase ii trial of human recombinant interleukin-2 administered as a 4-day continuous infusion for children with refractory neuroblastoma, non-hodgkin’s lymphoma, sarcoma, renal cell carcinoma, and Malignant melanoma. A childrens cancer group study. Cancer. (1995) 75:2959–65. doi: 10.1002/1097-0142(19950615)75:12<2959::aid-cncr2820751225>3.0.co;2-r 7773948

[142] Rosso R Sertoli MR Queirolo P Sanguineti O Barzacchi MC Mariani GL. An outpatient phase I study of a subcutaneous interleukin-2 and intramuscular alpha-2a-interferon combination in advanced Malignancies. Ann Oncol. (1992) 3:559–63. doi: 10.1093/oxfordjournals.annonc.a058261 1498078

[143] Little RF Pluda JM Wyvill KM Rodriguez-Chavez IR Tosato G Catanzaro AT. Activity of subcutaneous interleukin-12 in aids-related kaposi sarcoma. Blood. (2006) 107:4650–7. doi: 10.1182/blood-2005-11-4455 PMC1475826 16507779

[144] Little RF Aleman K Kumar P Wyvill KM Pluda JM Read-Connole E. Phase 2 study of pegylated liposomal doxorubicin in combination with interleukin-12 for aids-related kaposi sarcoma. Blood. (2007) 110:4165–71. doi: 10.1182/blood-2007-06-097568 PMC2234790 17846226

[145] Stinson JA Sheen A Momin N Hampel J Bernstein R Kamerer R. Collagen-anchored interleukin-2 and interleukin-12 safely reprogram the tumor microenvironment in canine soft-tissue sarcomas. Clin Cancer Res. (2023) 29:2110–22. doi: 10.1158/1078-0432.Ccr-23-0006 PMC10239368 37014656

[146] Qiu Y Su M Liu L Tang Y Pan Y Sun J. Clinical application of cytokines in cancer immunotherapy. Drug Des Devel Ther. (2021) 15:2269–87. doi: 10.2147/dddt.S308578 PMC8166316 34079226

[147] Propper DJ Balkwill FR. Harnessing cytokines and chemokines for cancer therapy. Nat Rev Clin Oncol. (2022) 19:237–53. doi: 10.1038/s41571-021-00588-9 34997230

[148] Recine F Vanni S Bongiovanni A Fausti V Mercatali L Miserocchi G. Clinical and translational implications of immunotherapy in sarcomas. Front Immunol. (2024) 15:1378398. doi: 10.3389/fimmu.2024.1378398 38983859 PMC11231074

[149] Teillaud JL Houel A Panouillot M Riffard C Dieu-Nosjean MC. Tertiary lymphoid structures in anticancer immunity. Nat Rev Cancer. (2024) 24:629–46. doi: 10.1038/s41568-024-00728-0 39117919

[150] Sonoda H Iwasaki T Ishihara S Mori T Nakashima Y Oda Y. Impact of tertiary lymphoid structure on prognosis and tumor microenvironment in undifferentiated pleomorphic sarcoma. Cancer Sci. (2025) 116:1464–73. doi: 10.1111/cas.70018 PMC12044655 40007136

[151] Wang XX Liu YP Lu Y Wu LH Ren JY Ji H. Identifying specific tls-associated genes as potential biomarkers for predicting prognosis and evaluating the efficacy of immunotherapy in soft tissue sarcoma. Front Immunol. (2024) 15:1372692. doi: 10.3389/fimmu.2024.1372692 38720884 PMC11076739

[152] Gu HY Lin LL Zhang C Yang M Zhong HC Wei RX. The potential of five immune-related prognostic genes to predict survival and response to immune checkpoint inhibitors for soft tissue sarcomas based on multi-omic study. Front Oncol. (2020) 10:1317. doi: 10.3389/fonc.2020.01317 32850416 PMC7396489

[153] Pan R Pan F Zeng Z Lei S Yang Y Yang Y. A novel immune cell signature for predicting osteosarcoma prognosis and guiding therapy. Front Immunol. (2022) 13:1017120. doi: 10.3389/fimmu.2022.1017120 36189307 PMC9515362

[154] Subramanian A Nemat-Gorgani N Ellis-Caleo TJ van IDGP Sears TJ Somani A. Sarcoma microenvironment cell states and ecosystems are associated with prognosis and predict response to immunotherapy. Nat Cancer. (2024) 5:642–58. doi: 10.1038/s43018-024-00743-y PMC11058033 38429415

[155] Vanni S Fausti V Fonzi E Liverani C Miserocchi G Spadazzi C. Unveiling the genomic basis of chemosensitivity in sarcomas of the extremities: an integrated approach for an unmet clinical need. Int J Mol Sci. (2023) 24:6926. doi: 10.3390/ijms24086926 37108089 PMC10138892

